# Development of the “Treatment beliefs in knee and hip OsteoArthritis (TOA)” questionnaire

**DOI:** 10.1186/s12891-017-1762-3

**Published:** 2017-09-19

**Authors:** Ellen M. H. Selten, Johanna E. Vriezekolk, Henk J. Schers, Marc W. Nijhof, Willemijn H. van der Laan, Roelien G. van der Meulen-Dilling, Rinie Geenen, Cornelia H. M. van den Ende

**Affiliations:** 1 Department of Rheumatology, Sint Maartenskliniek, Sint Maartenskliniek, P.O. Box 9011, 6500 GM Nijmegen, The Netherlands; 2 Department of Primary and Community Care, Radboud University Nijmegen Medical Center, Nijmegen, The Netherlands; 3 Department of Orthopedics, Sint Maartenskliniek, Nijmegen, The Netherlands; 4 Department of Rheumatology, Sint Maartenskliniek, Woerden, The Netherlands; 5 Physical Therapy and Manual Therapy Velperweg Partnership, Arnhem, The Netherlands; 6 Department of Psychology, Utrecht University, Utrecht, The Netherlands

**Keywords:** Osteoarthritis, Knee, Hip, Treatment beliefs, Measurement instrument

## Abstract

**Background:**

Use of conservative treatment modalities in osteoarthritis (OA) is suboptimal, which appears to be partly due to patients’ beliefs about treatments. The aim of this study was to develop a research instrument assessing patients’ beliefs about various treatment modalities of hip and knee OA: the ‘Treatment beliefs in OA (TOA) questionnaire’.

**Methods:**

The item pool that was retrieved from interviews with patients and healthcare providers comprised beliefs regarding five treatment modalities: physical activity, pain medication, physiotherapy, injections and arthroplasty. After an extensive selection procedure, a draft questionnaire with 200 items was constructed. Descriptive analyses and exploratory factor analyses with oblique rotation were conducted for each treatment modality separately to decide upon the final questionnaire. Internal consistency and test-retest reliability were determined.

**Results:**

The final questionnaire comprised 60 items. It was completed by 351 patients with knee or hip OA. Each of the five treatment modalities yielded a two factor solution with 37% to 51% explained variance and high face validity. Factor I included ‘positive treatment beliefs’ and factor II ‘negative treatment beliefs’. Internal consistency (Cronbach α’s from 0.72 to 0.87) and test-retest reliability (i.e. intraclass correlation coefficient from 0.66–0.88; standard error of measurement from 0.06–0.11) were satisfactory to good.

**Conclusions:**

The TOA questionnaire is the first questionnaire assessing positive and negative treatment beliefs regarding five treatment modalities for knee and hip OA. The instrument will help to understand whether and to what extent treatment beliefs influence treatment choices.

**Electronic supplementary material:**

The online version of this article (10.1186/s12891-017-1762-3) contains supplementary material, which is available to authorized users.

## Background

Osteoarthritis (OA) of the knee and hip causes pain, stiffness and decreased physical functioning [1]. Because OA cannot be cured, treatment is directed towards the reduction of symptoms, improvement of quality of life, and prevention of progression. Treatment options can be classified into conservative treatment modalities, such as lifestyle education, pain medication and physiotherapy, and surgical treatment modalities, such as an arthroplasty and osteotomy [1].

Several national and international recommendations and guidelines for the management of hip and knee OA recommend that patients first are provided with conservative treatment options, and that they are referred to surgical treatment only when conservative treatment does not lead to adequate pain relief and functional improvement [2–4]. However, in clinical practice, health care utilisation is suboptimal in terms of underutilisation of conservative treatment modalities [5–7] and an increased use of surgical treatment modalities [8]. This is undesirable because surgery does not always result in good outcomes and pain reduction [9, 10] and may lead to higher health care costs.

Amongst others, a possible pathway to optimise the imbalanced use of treatment options is through understanding patients’ beliefs about treatment modalities of knee and hip OA [11, 12]. Patients’ beliefs influence health-related behaviour as postulated by health beliefs models, such as the Theory of Planned Behaviour [13]. Previous research found that patients’ beliefs about the efficacy and safety of medication influence both their decision to take medication and their preference for the type of medication [14]. Moreover, it has been suggested that treatment choices can be better predicted when beliefs about multiple treatment options are assessed, instead of assessing beliefs about a single treatment option [15]. Therefore, identifying patients’ beliefs about various treatment modalities of OA may help to increase the understanding of treatment decisions.

At present, little is known about how and to what extent patients’ beliefs about treatment modalities of knee and hip OA influence patients’ treatment choices. Previous qualitative studies indicate that many considerations such as patients’ beliefs about the effectiveness and side-effects of the treatment may play a role in their treatment choice [16–18]. While qualitative studies are ideal to get an encompassing overview of all possible determinants of treatment choices, a measurement instrument based on self-reports is needed to get insight into the relative importance of treatment beliefs in the one patient as compared to the other. Therefore, guided by the results of these qualitative studies, a self-report instrument is needed in order to be able to systematically assess patients’ beliefs about treatment modalities for knee and hip OA. This instrument can be used in research to examine to what extent patients’ treatment beliefs contribute to the patients’ decision making process, and ultimately help to understand why conservative treatment modalities are underused in the management of knee and hip OA.

In the context of knee and hip OA, no questionnaire is available that comprehensively assesses patients’ beliefs about both surgical and conservative treatment modalities. Existing questionnaires assess fears and beliefs related to the consequences of knee OA [19] and expectations about the role of their physician in the management of knee OA [20]. Existing self-report instruments about treatment beliefs refer to low back pain [21], medicines [22] and surgery [15]. Therefore, the aim of the current study was to develop a questionnaire to assess patients’ beliefs about treatment modalities of knee and hip OA: the TOA (Treatment beliefs in OsteoArthritis) questionnaire, and to examine its factorial structure, internal consistency and test-retest reliability.

## Methods

### Development of item pool

For the development of a first draft of the TOA questionnaire an elaborated process was undertaken to generate and select items based on the findings of three previous studies among patients with knee and hip OA in the Netherlands. These were two qualitative studies [17, 23] on treatment beliefs in patients and healthcare providers and a concept mapping study [24] to define the most important themes. A total of 2207 statements reflecting beliefs about treatment modalities of knee or hip OA (which could be potentially included as items in the TOA questionnaire) were extracted from the interviews. Items were selected from 4 major themes originated from the concept mapping study: ‘contextual barriers’ (e.g. the healthcare system), disadvantages (e.g. risks), treatment outcomes (e.g. physical functioning) and ‘outcomes for personal life’ (e.g. activities of daily living) [24].

The draft TOA questionnaire consisted of five modules, based on five treatment options recommended in the ‘stepped care strategy’ for knee and hip OA in the Netherlands [3]: physical activities, pain medication, physiotherapy, injections and arthroplasty. We aimed for a feasible set of approximately 50 items to include in each module in the draft version of the TOA questionnaire. In a careful and thorough consensus process, as described previously [24], all 2207 statements about specific treatment modalities derived from the interviews were reduced by a project group comprising researchers and health professionals including medical specialists. The selection procedure comprised several steps. For each step, cut-off points were developed to reach a representative set of 51 general items (Additional file 1). Two patient partners assessed this set of 51 items for its representativeness and comprehensiveness. In the next step, all 51 items were assessed for its applicability to each of the 5 treatment modalities. For instance, the item ‘the treatment may cause an infection’ was applicable for the treatment modality injections and arthroplasty, but not for physical activity, pain medication and physiotherapy. If applicable, the item was included in the module. The final draft version of the TOA questionnaire comprised 200 items, distributed over 5 modules: physical activities (41 items), pain medication (37 items), physiotherapy (42 items), injections (41 items) and arthroplasty (39 items). A 5-point Likert scale with scoring options ranging from 1 to 5 was chosen, labelled from ‘disagree’ to ‘agree’ in order to avoid end-aversion bias (i.e. avoiding absolute statements as ‘completely disagree’ and ‘completely agree’ to overcome the reluctance of some people to use extreme categories of a scale) [25]. The TOA questionnaire was developed in Dutch. An English translation of the items can be found in Additional file 2.

### Pilot testing

The draft TOA questionnaire was pilot tested in a stepwise way. The first draft of the questionnaire was tested by five researchers and health professionals. Subsequently, ten patients were recruited via a primary care physiotherapy practice in the Northern part of the Netherlands. Besides the clinical diagnosis of knee or hip OA, no other inclusion criteria were required. Patients were asked to fill out the questionnaire at home and to make notes if they thought a question was difficult to understand. Hereafter, the researcher contacted the patient for a telephone interview. All items of the questionnaire were discussed, using the probing method for pilot-testing whether the patient understood the items, whether items were interpreted according to their intended meaning, and whether the length of the questionnaire was considered acceptable [26, 27]. Including patients for the pilot test was stopped after 10 interviews because no new information emerged from interviewing the last two patients (data saturation). Based on the results of the pilot test, minor alterations were made in the instructions and lay-out.

### Patients and measures

Two different samples were recruited for this study. The first sample was recruited to examine the factor structure and internal consistency of the TOA questionnaire; the second sample was recruited to examine the test-retest reliability of the TOA questionnaire.

#### Sample 1: Factor structure and internal consistency

Eligible patients who visited the department of Rheumatology of the Sint Maartenskliniek in 2013–2014 (*n* = 600, randomly selected from the electronic patient record system) or the department of Orthopaedics in June–August 2015 (*n* = 240, consecutively), who were clinically diagnosed with knee or hip OA, and were aged ≥18 received an information letter and informed consent form. Assuming a number of 4–10 participants per item and 51 unique items, a sample size of at least 204 patients was needed to perform the factor analysis [26]. These patients filled out the TOA questionnaire once. In addition, demographic and clinical characteristics were collected: body mass index (BMI), duration of OA symptoms, affected joint(s), comorbidities (question 70 from the DUTCH-AIMS2 [28]), treatment use, and the Dutch version of the Western Ontario and McMaster Universities Arthritis LK3.1 Index (WOMAC). The WOMAC is a health status measure assessing the dimensions of pain, stiffness and function in patients with OA of the hip/knee [29].

#### Sample 2: Test-retest reliability analysis

Patients were consecutively selected from a larger study sample with similar eligibility criteria as sample 1. To determine test-retest reliability by calculating an ICC of 0.8 with a 95% confidence interval ± 0.1 using 2 repeated measurements, 50 respondents are required [26]. Eligible patients of the department of Rheumatology of the Sint Maartenskliniek in 2015–2016 (*n* = 39) or the department of Orthopaedics in September 2015–September 2016 (*n* = 41) were randomly selected from the electronic patient record system. Patients were invited to fill out the final TOA questionnaire twice, with a 2 weeks interval. The first 50 respondents who sent the questionnaire back were included in the analysis, to keep the time between the first and second measurement close to the aimed interval of 2 weeks.

The medical ethical board of the Radboud University Medical Center, Nijmegen concluded that the Dutch Medical Research Involving Human Subjects Act did not apply to this study (protocol number: 2015–1772 for sample 1 and protocol number 2016–2605 for sample 2).

### Statistical analyses

Because the TOA questionnaire comprises five treatment modalities, we aimed for a brief set of items per treatment module. Therefore, rigorous item reduction and exploratory factor analysis were used to design the final TOA questionnaire. This was conducted per module in three consecutive steps: initial item reduction, factor analysis and further refinement. Furthermore, internal consistency (Cronbach’s alpha) and the test-retest reliability of the final TOA questionnaire were examined per module.

#### Step 1: Initial item reduction

Items were considered to be deleted if: a) missing values were >15%; b) >50% of patients scored 1 (disagree) or 5 (agree) on an item (floor or ceiling effect); c) skewness of the item was >1; d) inter-item correlations were >.80 (in this case one of the redundant paired items was considered for deletion) [25].

#### Step 2: Factor analysis and internal consistency

Exploratory factor analyses were conducted for each modality separately to examine the dimensionality of the TOA questionnaire. First, exploratory factor analysis without rotation was used to determine the initial numbers of factors. This was determined by two researchers (JV and ES) by visual inspection of the scree plot, percentages of explained variance (>5%) and eigenvalues >1 [30]. Thereafter, for each module exploratory factor analysis with oblique (direct oblimin) rotation was conducted for 2-factor to 4-factor solutions. Oblique rotation was chosen because it allowed the extracted factors to be correlated. To select the most salient items two criteria were used: only items with factor loadings ≥0.45 were retained, and items with cross loadings on more than one factor within 0.3 of the primary loading were dropped because of inadequate discrimination [31]. The final number of factors per module was determined by the project group based on factor interpretability and revealed a 2-factor solution for each module.

#### Step 3: Further refinement

Guided by the results of a previous concept mapping study [24], items were further considered for deletion. Briefly, in this concept mapping study, 36 patients sorted the 51 items (each item printed on a card) from the TOA questionnaire into piles with a similar meaning; subsequently hierarchical cluster analysis yielded a 15-cluster solution that was grouped in 4 higher-order categories and 2 overarching categories. The following additional rules (set by the project group) were applied for further item reduction per module:

1. Each factor should contain preferably a maximum of 1 item per cluster
2. If more than 1 item per cluster loaded on the factor, the item with the highest factor loading was retained
3. If internal consistency of a factor, as assessed with Cronbach’s alpha, dropped below .70, the item with the next highest factor loading was retained.
4. If internal consistency of the factor, as assessed with Cronbach’s alpha, was still < .70, an item with the next highest factor loading from another cluster was added.

Lastly, a final two-factor factor analysis with the remaining items per module was conducted. Cronbach’s alpha was calculated for each factor, and Pearson’s correlation coefficients were calculated between factor I and factor II per module.

#### Test-retest reliability

Because each method to assess test-retest reliability of a questionnaire has its advantages and disadvantages and is difficult to interpret without other methods [32], multiple methods were used to assess the test-retest reliability of the TOA questionnaire. Test-retest reliability was determined by 1) Intraclass correlation coefficients (ICCs) (two-way mixed effects model, measuring consistency of individual differences) [33]; 2) Limits of agreement (LoA); and 3) Standard errors of measurement (SEMs) of the whole model including systematic differences between repeated measures. Scale scores of the TOA questionnaire were calculated by summation of the items for each factor. When a respondent had ≤25% missing items on a subscale, these missing items were substituted by the respondent’s mean sum score on the subscale. When a respondent had >25% missing items on a subscale, these were taken into account as missing values in the analysis. Unstandardised scores for each subscale per module were used for calculating ICCs and the LoA. For calculating the SEM, standardised scores were calculated (raw total score of subscale / total items on subscale). Thus, total scores on each subscale were comparable on a scale from 1 to 5. ICCs range from 0 to 1, whereby 1 reflects perfect reliability. In general, ICCs ≥0.70 are considered acceptable [34]. LoA were calculated with the following formula: mean difference ± 1.96 x SD_difference_. SEM of the whole model including systematic variation of repeated measures was calculated for each subscale with: √(σ^2^_*m*_ + σ^2^_*residual*_). Where σ^2^_*m*_ *=* variance between the two repeated measures; and σ^2^_*residual*_ *=* variance of the residual (“error”). A smaller SEM reflects better test-retest reliability [26].

All analyses were performed using STATA 13.1.

## Results

### Participants

#### Sample 1

Of 840 invited patients, 351 filled out the TOA questionnaire and provided informed consent (response rate: 41.8%). Eighty-two patients indicated they did not want to participate in the study. Some patients provided a reason for non-participation, e.g.: no knee or hip OA (*n* = 25), not wanting to (*n* = 5), comorbidities (*n* = 4), not satisfied about care provided by the hospital (*n* = 3). Ten patients who did fill out the questionnaire were excluded because they did not provide informed consent. Three patients did not fill out the additional questionnaire assessing demographic and clinical characteristics but were included in the factor analysis. Table 1 shows the demographic and clinical characteristics of the study sample.

**Table 1:** Characteristics of the study sample (*N*=348^a^)

Demographic characteristics	
Age (years), mean (SD)	62.8 (12.3)
Gender (female), *n* (%)	217 (63.1)
Married or cohabiting, *n* (%)	273 (79.4)
Currently employed, *n* (%)^*b*^	145 (44.1)
Education level, *n* (%)^*c*^	
Low	62 (18.0)
Middle	175 (50.9)
High	107 (31.1)
Clinical characteristics	
Body Mass Index (BMI) (kg/m^2^), n(%)	
Normal weight (BMI <25)	106 (31.0)
Overweight (BMI 25–30)	146 (42.7)
Obese (BMI > 30)	90 (26.3)
Duration of OA symptoms (years), mean (SD)^*b*^	11.1 (9.8)
Affected joint(s), *n* (%)	
Hip	82 (24)
Knee	169 (49.5)
Hip and knee	91 (26.5)
Comorbidities, n(%)^*d*^	
No comorbidities	141 (40.8)
High blood pressure	97 (28.0)
Heart disease	39 (11.2)
Diabetes	27 (7.8)
Lung disease	28 (8)
Other	42 (12)
Previous or current treatments for OA, *n* (%)^*d*^	
Pain medication	291 (85.3)
Physiotherapy	234 (68.6)
Injections	133 (39.0)
Surgery	112 (32.8)
WOMAC^e^ (Likert scale 0–4), unstandardized mean (SD), theoretical range	
Pain^*b*^	10.0 (4.5), 0–20
Stiffness	4.5 (2.0), 0–8
Functioning	32.4 (14.8), 0–68
Total (sum score)^*b*^	46.7 (20.0), 0–96

^*a*^ *3 respondents did not fill out these questions*

^b^Missing values > 5%: Currently employed = 6%; Duration of OA symptoms = 7%; WOMAC subscale pain = 5%, WOMAC total (sum score) = 8%

^c^Low = no education, primary school, lower vocational education; Middle = secondary school, middle vocational education; High = higher vocational education, university

^d^More than 1 answer possible

^e^Western Ontario and McMaster Universities Arthritis Index. Higher scores reflect worse pain, stiffness and functioning

#### Sample 2

Of the 80 patients who were invited to fill out the final TOA questionnaire twice, 67 patients returned the questionnaire (response rate: 83.8%). The first 50 respondents who sent the questionnaire back were included in the analysis, to keep the time between the first and second measurement close to the aimed interval of 2 weeks (Mean time interval = 13 days, SD = 2.5, range = 6–18). The mean age of sample 2 was 63.8 years (SD = 10.5), and 56% was female.

### Step 1: Initial item reduction

Item reduction resulted in dropping 9, 8, 8, 2, and 4 items respectively in modules 1 to 5. Two pairs of items in module 4 had a correlation of 0.81 and 0.82, but because the items reflected different contents, none of the items were deleted.

### Step 2: Factor structure and internal consistency

Based on exploratory factor analyses and interpretability, a two-factor solution for each module was obtained. Respectively 8, 12, 16, 18, 14 items were dropped because of factor loadings ≥0.45 or cross loadings <0.3 in module 1 to 5 (Additional file 2).

### Step 3: Further refinement

After the previous steps of item reduction, a total of 24, 17, 18, 22 and 21 items remained for module 1 to 5 respectively). After the third step of refinement, 13 items remained for module 1 (physical activities), 12 items for module 2 (pain medication), 9 items for module 3 (physiotherapy), 12 items for module 4 (injections), and 14 items for module 5 (arthroplasty). After this last round of item reduction, a final 2-factor factor analysis with oblique (direct oblimin) rotation was performed for all modules. The explained percentage of variance per module ranged from 37% to 51%, Cronbach’s alpha’s of the final TOA questionnaire ranged from .72 to .87, and correlations between factors ranged from −.03 to −.51 (Table 2).

**Table 2:** Factor loadings, eigenvalues, percentage of explained variance and Cronbach’s alpha for the final TOA questionnaire

Module 1: Physical activities		
*Items*	*Factor loadings*	
* M1 = Module 1, Q = Question number (see* Additional file 1 *)*	*Factor I*	*Factor II*
M1Q32: I learn to deal with my symptoms better by doing physical activities	.77	
M1Q19: I can do household chores better by doing physical activities	.72	
M1Q22: Doing physical activities produces good results at my age	.71	
M1Q33: I can do my job better by doing physical activities	.70	
M1Q39: I can tailor doing physical activities to my goals	.63	
M1Q10: I can postpone surgery by doing physical activities	.53	
M1Q14: I can do physical activities together with others	.48	
M1Q8: The only way to reduce my OA symptoms is by doing physical activities	.47	
M1Q20: I enjoy doing physical activities	.45	
M1Q24: By doing physical activities I will overload my knee/hip		.72
M1Q23: I think that doing physical activities causes pain		.66
M1Q7: I think that doing physical activities involves risks		.65
M1Q28: I am scared to do physical activities		.55
Eigenvalue	4.03	1.06
Percentage of variance	31%	8%
Cronbach’s Alpha	.84	.79
Correlation between factors	−.51	
Module 2: Pain medication		
*Items*	*Factor loadings*	
* M2 = Module 2, Q = Question number (see* Additional file 1 *)*	*Factor I*	*Factor II*
M2Q10: I can move more freely by using painkillers	.80	
M2Q18: I can do household chores better by using painkillers	.76	
M2Q2: My quality of life increases by using painkillers	.74	
M2Q20: Using painkillers produces good results at my age	.73	
M2Q9: I can postpone surgery by using painkillers	.50	
M2Q15: Using painkillers is harmful to my health		.70
M2Q6: I think that using painkillers involves risks		.67
M2Q11: I think that painkillers have side-effects		.64
M2Q28: I think that using painkillers is invasive		.52
M2Q25: I am scared to use painkillers		.47
M2Q19: I think that using painkillers leads to habituation		.38
M2Q22: By using painkillers I will overload my knee/hip		.35
Eigenvalue	2.74	2.00
Percentage of variance	23%	17%
Cronbach’s Alpha	.82	.72
Correlation between factors	−.14	
Module 3: Physiotherapy		
*Items*	*Factor loadings*	
* M3 = Module 3, Q = Question number (see* Additional file 1 *)*	*Factor I*	*Factor II*
M3Q23: Doing physiotherapy produces good results at my age	.86	
M3Q20: I can do household chores better by physiotherapy	.85	
M3Q34: I can do my job better by physiotherapy	.76	
M3Q3: My quality of life increases by physiotherapy	.71	
M3Q22: I need to actively get going with physiotherapy myself	.60	
M3Q10: I can postpone surgery by physiotherapy	.52	
M3Q25: By physiotherapy I will overload my knee/hip		.71
M3Q24: I think that physiotherapy causes pain		.70
M3Q7: I think that physiotherapy involves risks		.62
Eigenvalue	3.49	1.09
Percentage of variance	39%	12%
Cronbach’s Alpha	.86	.74
Correlation between factors	−.36	
Module 4: Injections		
*Items*	*Factor loadings*	
* M4 = Module 4, Q = Question number (see* Additional file 1 *)*	*Factor I*	*Factor II*
M4Q33: I can do my job better by an injection	.89	
M4Q20: I can do household chores better by an injection	.88	
M4Q3: My quality of life increases by an injection	.76	
M4Q30: An injection gives quick results	.76	
M4Q10: I can postpone surgery by an injection	.62	
M4Q31: I think that an injection can be repeated	.47	
M4Q24: By an injection I will overload my knee/hip		.62
M4Q15: I am becoming dependent on an injection		.61
M4Q34: An injection damages my knee/hip		.60
M4Q32: I think that an injection is invasive		.54
M4Q7: I think that an injection involves risks		.48
M4Q40: An injection takes a lot of my time		.38
Eigenvalue	3.54	1.78
Percentage of variance	30%	15%
Cronbach’s Alpha	.87	.72
Correlation between factors	−.11	
Module 5: Arthroplasty		
*Items*	*Factor loadings*	
* M5 = Module 5, Q = Question number (see* Additional file 1 *)*	*Factor I*	*Factor II*
M5Q4: My pain increases by a joint replacement	−.61	
M5Q9: My knee/hip deteriorates faster by a joint replacement	−.51	
M5Q10: I can move more freely after a joint replacement	.78	
M5Q17: I can do household chores better after a joint replacement	.77	
M5Q18: A joint replacement produces good results at my age	.81	
M5Q21: More people with OA choose to do a joint replacement	.43	
M5Q29: I think that a joint replacement can be repeated	.45	
M5Q38: I think that an artificial joint lasts a long time	.44	
M5Q1: I think a joint replacement is painful		.51
M5Q23: I think an artificial joint carries the chance of an infection		.57
M5Q24: I think a joint replacement carries the chance of an infection		.67
M5Q27: A joint replacement takes up my energy		.53
M5Q30: I think that a joint replacement is invasive		.62
M5Q37: A joint replacement takes a lot of my time		.60
Eigenvalue	3.13	2.14
Percentage of variance	22%	15%
Cronbach’s Alpha	.81	.75
Correlation between factors	−.03	

### Factor interpretation

For each module, the first factor reflected positive beliefs about the treatment, such as health benefits and perceived advantages (e.g. “I learn to deal with my symptoms better by the treatment”). The second factor reflected negative beliefs about the treatment, such as treatment risks and disadvantages (e.g. “I think that the treatment involves risks”). Therefore, for each module, Factor I was labelled ‘positive treatment beliefs’ and Factor II was labelled ‘negative treatment beliefs’. For each subscale in each module of the TOA questionnaire, a sum score can be calculated whereby a higher sum score on subscale I reflects more positive treatment beliefs and a higher sum score on subscale II reflects more negative treatment beliefs. Scorings on the questions M5Q4 and M5Q9 should be reversed. Missing item scores on a subscale are replaced with the mean of the other items; when more than 25% of the items are missing, the subscale score is not valid.

Table 2 presents the factor loadings, eigenvalues, percentages of explained variance, Cronbach’s alpha for each factor per treatment module, and the correlations between factors per treatment module.

### Test-retest reliability

Table 3 shows 3 different measures of test-retest reliability for each subscale of the TOA questionnaire. Considering the moderate to high ICCs (0.66–0.88) and small SEM (0.06–0.11) obtained for all subscales, test-retest reliability of the TOA questionnaire was satisfactory.

**Table 3:** Intraclass correlation coefficient (ICC), mean difference between repeated measures, limits of agreement (LoA), and standard error of measurement (SEM) including systematic differences between repeated measures and error variance

Module	Subscale	ICC	Mean difference	LoA	SEM
Physical activity	positive *9 items*	.88	−.44	−7.65; 6.78	.06
	negative *4 items*	.74	.81	−4.60; 6.23	.10
Pain medication	positive *5 items*	.80	.19	−6.44; 6.82	.09
	negative *7 items*	.83	.59	−6.82; 8.00	.08
Physiotherapy	positive *6 items*	.88	.16	−6.35; 6.67	.08
	negative *3 items*	.72	−0.40	−4.81; 4.01	.11
Injections	positive *6 items*	.88	0.05	−5.85; 5.96	.07
	negative *6 items*	.83	.45	−5.06; 5.96	.07
Arthroplasty	positive *8 items*	.66	−.20	−9.14; 8.75	.08
	negative *6 items*	.77	.45	−5.52; 6.41	.07

## Discussion

The TOA questionnaire is the first questionnaire assessing treatment beliefs regarding surgical and conservative (physical activities, pain medication, physiotherapy, injections) modalities for knee and hip OA. The TOA questionnaire comprises five treatment modalities with each a positive and negative subscale. Each part of the questionnaire can be used independently, so beliefs regarding either one or multiple treatment modalities can be measured. A main strength of this study was the design used to generate the items. For the selection of items, we used two previous in-depth interview studies in which both patients and healthcare providers were asked about their beliefs and views regarding treatment modalities for knee and hip OA [17, 23]. The item pool was generated very carefully in several consensus rounds by the project team, and selected items were assessed by patients. As a result, based on the perspective of patients and professionals, we developed a questionnaire to comprehensively assess both positive and negative treatment beliefs in knee and hip OA. The qualitative approach will have contributed to the face validity. The internal consistency and test-retest reliability of the TOA questionnaire were satisfactory to good. Confirmatory factor analysis and replication of clinimetric properties in other samples as well as validation studies such as studies examining the association with actual treatment choices are needed in order to fully establish the validity and reliability of the TOA questionnaire.

The 2-factor structure reflected individual differences in positive and negative beliefs about treatment modalities. Similarly to existing generic questionnaires about medication (Beliefs about Medicines Questionnaire (BMQ) [22]) and surgery (Beliefs about Surgery Questionnaire (BSQ) [15]), the TOA questionnaire assesses negative treatment beliefs as a distinct dimension. In contrast to the BMQ and BSQ, the TOA questionnaire also assesses positive beliefs about treatment modalities. The small correlations between the factors – especially for the modules pain medication, injections and arthroplasty – show that positive and negative beliefs are not the opposite poles of a single dimension. This indicates the importance of measuring both patients’ negative and positive treatment beliefs in order to fully understand patients’ treatment preferences.

The TOA questionnaire can primarily be used as a research tool to assess individual differences in treatment beliefs and to examine to what extent treatment beliefs influence treatment choices in OA. Previous research showed that patients with knee or hip OA differ in their willingness to undergo surgery, and that this difference might be due to individual differences in sex, ethnicity, socioeconomic status [35], severity, age and income [36]. In interaction with, and in addition to these sociodemographic characteristics, treatment beliefs will likely play a role in treatment choices, specifically in suboptimal use of conservative treatment modalities. Previous studies have demonstrated the practice variation in primary care settings with regard to diagnostic procedures and referrals [37, 38], and that referrals by the GP to other disciplines are associated with patients’ preferences [39]. This suggests that besides organisational and healthcare provider-related factors, patients’ treatment beliefs should be taken into account, in order to choose a treatment that fits best to the patient’s individual situation and preferences. In clinical practice, individual scores at the TOA questionnaire could be used as an input for shared decision making. However, users should be aware that the item pool reflects a restricted number of items that predominantly reflect individual differences. To get an encompassing overview of treatment beliefs that may be important for an individual patient, it is better to use all statements from a previous concept mapping study [24] which represent a wide range of potential benefits and barriers.

Some limitations of the study need to be addressed. Firstly, our findings in a secondary care sample cannot be generalised to other samples or settings without empirical replication. With respect to external validity, cross-cultural validation studies are needed to examine whether the TOA questionnaire is valid to use in other languages and cultures than Dutch. Also other aspects of validity need to be more extensively evaluated, such as construct validity and criterion validity. In new samples, the structural validity of the TOA questionnaire could be further evaluated by using confirmatory factor analyses to verify whether the factor structure is replicated and Item-Response Theory to improve the precision of the measurement instrument [26]. On average, our sample reported moderate OA complaints in terms of pain, stiffness and functioning [29, 40]. Future research needs to examine the robustness of the factor structure of the TOA in other samples. Secondly, the response rate for sample 1 in this study was 42%, which could indicate a response bias. The questionnaire was quite long, which might have been burdensome for patients. The response rate, however, is comparable to other studies in knee or hip OA [41]. Moreover, 351 respondents filled in the questionnaire, which is sufficient for a factor analysis [26]. Thirdly, patients’ involvement in the item reduction process for the TOA questionnaire was limited to an assessment of the comprehensiveness and completeness of the items by two patients and an extensive pilot-test in 10 primary care patients. However, items for the TOA questionnaire were selected in a careful and thorough process to enhance the validity of the questionnaire.

## Conclusions

The TOA questionnaire assesses positive and negative treatment beliefs of patients with knee or hip OA about 5 treatment modalities: physical activities, pain medication, physiotherapy, injections and arthroplasty. Initial analyses of the clinimetric properties of the TOA questionnaire are promising. The questionnaire can be used in research to clarify treatment choices. Future research should assess the validity and reliability of the TOA questionnaire in other OA samples, and should verify whether treatment beliefs in interaction with other variables influence intended and actual treatment choices.

## Additional files

Additional file 1: Procedure that was used for selecting a representative set of items from statements resulting from interviews. Description: Procedure that was used for selecting a representative set of items from statements resulting from interviews. (DOCX 22 kb)

Additional file 2: Initial factor loadings for items in the Treatment beliefs in OsteoArthritis Questionnaire in patients with knee and hip osteoarthritis (*N* = 351). Description: Initial factor loadings for items in the Treatment beliefs in OsteoArthritis Questionnaire. (DOCX 42 kb)

## Abbreviations

BMI: Body mass index
ICC: Intraclass correlation coefficient
LoA: Limits of agreement
OA: Osteoarthritis
SD: Standard deviation
SEM: Standard error of measurement
TOA: Treatment beliefs in osteoarthritis
WOMAC: Western ontario and McMaster universities arthritis index
