# Supplementary material for: Development of the “Treatment beliefs in knee and hip OsteoArthritis (TOA)” questionnaire
Source: BMC Musculoskelet Disord. 2017 Sep 19;18:402. doi: 10.1186/s12891-017-1762-3 (PMC5606114; doi:10.1186/s12891-017-1762-3)
Supplement: Supplementary file 1 — Procedure that was used for selecting a representative set of items from statements resulting from interviews. Description: Procedure that was used for selecting a representative set of items from statements resulting from interviews. (DOCX 22 kb) [file 12891_2017_1762_MOESM1_ESM.docx]

*Additional file 1: Procedure that was used for selecting a representative set of items from statements resulting from interviews*

| Step | Name | Participants | Task |
| --- | --- | --- | --- |
| 1 | Extraction from interviews | Researcher 1 | All statements were extracted from the dataset with interviews. |
| 2 | Removal of duplication | Researcher 1 | Duplicated statements were removed from the set. |
| 3 | Selection round 1 | Researcher 1 + 2 | Two researchers independently evaluated statements to be retained. Each statement was assigned the label ‘yes’, ‘no’ or ‘?’, based on the following criteria^1^:  a) Generalizability of the statements to entire patient population  b) Wording of the statements (e.g. ambiguity, comprehensibility, abstractness, terminology) |
| 4 | Collation of statements | Researcher 1 + 2 | The retained statements of both researchers were collated. Statements that were labelled ‘yes’ by both researchers were included in the next step of reduction. Statements labelled by one or both researchers with ‘?’ were discussed until consensus was reached about a feasible number of statements to be judged by an expert panel. |
| 4 | Selection round 2 | Expert panel^2^ | Each member of the expert panel independently evaluated 1/3 of the statements (so each statement was assessed by 2 members), following the same criteria as described in step 3. The statements that were judged with a ‘yes’ by the majority of the expert panel were included in the final set of statements, whereby representativeness of the complete set of statements was safely guarded by the researchers. |
| 5 | Consensus meeting | Expert panel^2^ | The final set of statements was discussed with an expert panel for its representativeness and completeness. |
| 6 | Writing | Researcher 1 + 2 | The retained statements were written on cards; preferably in patients’ own words. If necessary, the wording and length of a statement was altered to fit the level of reading skills of a 12-year old^2^. |
| 7 | Patient check | Researcher 1 + (at least) 2 patients | Two patients evaluated the final set of statements based on its clarity and comprehensibility , and were given the opportunity to add statements if they missed any. |

^1^ Based on: Streiner DL, Norman GR. Health Measurement Scales. A practical guide to their development and use. 4th ed. New York: Oxford University Press Inc.; 2008.

^2^The expert panel consisted of 6 persons (co-authors): 2 researchers (others than Researcher 1 and 2), 2 medical specialists, a GP and a physiotherapist
