# Supplementary material for: Development of the “Treatment beliefs in knee and hip OsteoArthritis (TOA)” questionnaire
Source: BMC Musculoskelet Disord. 2017 Sep 19;18:402. doi: 10.1186/s12891-017-1762-3 (PMC5606114; doi:10.1186/s12891-017-1762-3)
Supplement: Supplementary file 2 — Initial factor loadings for items in the Treatment beliefs in OsteoArthritis Questionnaire in patients with knee and hip osteoarthritis (N = 351). Description: Initial factor loadings for items in the Treatment beliefs in OsteoArthritis Questionnaire. (DOCX 42 kb) [file 12891_2017_1762_MOESM2_ESM.docx]

Supplementary material 1: Initial factor loadings for items in the Treatment beliefs in OsteoArthritis Questionnaire in patients with knee and hip osteoarthritis (N=351).

M1Q1 = Module 1, Question 1. Rotated pattern matrix: exploratory factor analysis with direct oblimin rotation.

*Step 1: Item reduction before exploratory factor analysis based on visual inspection of the frequency tables and histograms, missing values, floor or ceiling effect, Skewness-Kurtosis, inter-item correlations >0.80.

**Step 2: Item reduction based on exploratory factor analysis (item loadings > = 0.45 were retained, and items with cross loadings on more than one factor within 0.3 of the primary loading were dropped)

***Step 3: Item reduction based on the maximum of 1 item per cluster (based on previous cluster analysis)

^ Initial factor loadings after step 1 (before step 2 and 3)

| Module 1: Physical exercises | | | | |
| --- | --- | --- | --- | --- |
|  | | | Initial factor loadings^ | |
| *Item pool* | *Brief description* | *Retained items* | *Factor I* | *Factor II* |
| M1Q1*** | I think that doing physical exercises is painful |  | 0.01 | 0.58 |
| M1Q2** | My thoughts about doing physical exercises is: “there’s no harm in trying” |  | 0.18 | 0.09 |
| M1Q3* | My quality of life increases by doing physical exercises |  | - | - |
| M1Q4*** | My pain increases by doing physical exercises |  | -0.07 | 0.65 |
| M1Q5*** | I can participate in social activities again by doing physical exercises |  | 0.70 | 0.05 |
| M1Q6** | I am more likely to overdo it by doing physical exercises |  | 0.32 | 0.47 |
| M1Q7 | I think that doing physical exercises involves risks | M1Q7 | 0.02 | 0.66 |
| M1Q8 | The only way to reduce my OA symptoms is by doing physical exercises | M1Q8 | 0.57 | 0.10 |
| M1Q9*** | My knee/hip deteriorates faster by doing physical exercises |  | -0.12 | 0.61 |
| M1Q10 | I can postpone surgery by doing physical exercises | M1Q10 | 0.57 | -0.04 |
| M1Q11*** | I can move more freely by doing physical exercises |  | 0.66 | -0.15 |
| M1Q12* | I can tailor physical exercises to what I am able to do |  | - | - |
| M1Q13*** | I think it has been proven that physical exercises reduce OA symptoms |  | 0.54 | -0.09 |
| M1Q14 | I can do physical exercises together with others | M1Q14 | 0.46 | 0.01 |
| M1Q15* | I can do physical exercises where and whenever I like |  | - | - |
| M1Q16* | Doing physical exercises is harmful to my health |  | - | - |
| M1Q17*** | I think that the effect of physical exercises lasts a long time |  | 0.58 | 0.04 |
| M1Q18* | It is easy to fit doing physical exercises into my daily life |  | - | - |
| M1Q19 | I can do household chores better by doing physical exercises | M1Q19 | 0.74 | 0.02 |
| M1Q20 | I enjoy doing physical exercises | M1Q20 | 0.45 | 0.05 |
| M1Q21* | I need to actively get going with physical exercises myself |  | - | - |
| M1Q22 | Doing physical exercises produces good results at my age | M1Q22 | 0.69 | -0.01 |
| M1Q23 | I think that doing physical exercises causes pain | M1Q23 | 0.06 | 0.79 |
| M1Q24 | By doing physical exercises I will overload my knee/hip | M1Q24 | -0.10 | 0.71 |
| M1Q25** | I have to pay (part of) the costs of physical exercises myself |  | 0.12 | 0.18 |
| M1Q26** | More people with OA choose to do physical exercises |  | 0.41 | 0.06 |
| M1Q27** | I am able to stop taking painkillers by doing physical exercises |  | 0.22 | -0.03 |
| M1Q28 | I am scared to do physical exercises | M1Q28 | 0.02 | 0.53 |
| M1Q29** | Doing physical exercises takes up my energy |  | 0.10 | 0.36 |
| M1Q30*** | Doing physical exercises gives quick results |  | 0.59 | 0.03 |
| M1Q31* | I think that physical exercises can be repeated |  | - | - |
| M1Q32 | I learn to deal with my symptoms better by doing physical exercises | M1Q32 | 0.76 | 0.04 |
| M1Q33 | I can do my job better by doing physical exercises | M1Q33 | 0.74 | -0.07 |
| M1Q34*** | Doing physical exercises damages my knee/hip |  | -0.11 | 0.64 |
| M1Q35** | I think that physical exercises are easy to perform |  | 0.43 | -0.05 |
| M1Q36*** | My pain lessens by doing physical exercises |  | 0.67 | -0.15 |
| M1Q37* | I value the advice of a healthcare provider about doing physical exercises |  | - | - |
| M1Q38*** | I think that doing physical exercises reduces inflammation |  | 0.56 | 0.02 |
| M1Q39 | I can tailor doing physical exercises to my goals | M1Q39 | 0.65 | 0.01 |
| M1Q40** | Doing physical exercises takes a lot of my time |  | 0.07 | 0.23 |
| M1Q41* | I am dismissive about doing physical exercises because I believe that OA is a fact of life |  | - | - |

| Module 2: Pain medication | | | | |
| --- | --- | --- | --- | --- |
|  | | | Initial factor loadings^ | |
| *Item pool* | *Brief description* | *Retained items* | *Factor I* | *Factor II* |
| M2Q1** | My thoughts about using painkillers is: “there’s no harm in trying” |  | 0.19 | -0.04 |
| M2Q2 | My quality of life increases by using painkillers | M2Q2 | 0.74 | -0.03 |
| M2Q3* | My pain increases by using pain medication |  | - | - |
| M2Q4*** | I can participate in social activities again by using painkillers |  | 0.74 | -0.04 |
| M2Q5** | I am more likely to overdo it by using painkillers |  | 0.32 | 0.36 |
| M2Q6 | I think that using painkillers involves risks | M2Q6 | -0.10 | 0.60 |
| M2Q7** | The only way to reduce my OA symptoms is by using painkillers |  | 0.43 | 0.03 |
| M2Q8** | My knee/hip deteriorates faster by using painkillers |  | -0.06 | 0.43 |
| M2Q9 | I can postpone surgery by using painkillers | M2Q9 | 0.51 | 0.06 |
| M2Q10 | I can move more freely by using painkillers | M2Q10 | 0.78 | 0.10 |
| M2Q11 | I think that painkillers have side-effects | M2Q11 | -0.01 | 0.54 |
| M2Q12*** | I think it has been proven that using painkillers reduce OA symptoms |  | 0.45 | 0.01 |
| M2Q13** | I am becoming dependent on taking painkillers |  | 0.34 | 0.45 |
| M2Q14** | I can us painkillers where and whenever I like |  | 0.10 | -0.13 |
| M2Q15 | Using painkillers is harmful to my health | M2Q15 | -0.12 | 0.64 |
| M2Q16** | I think that the effect of painkillers lasts a long time |  | 0.41 | -0.08 |
| M2Q17* | It is easy to fit using painkillers into my daily life |  | - | - |
| M2Q18 | I can do household chores better by using painkillers | M2Q18 | 0.76 | 0.03 |
| M2Q19 | I think that using painkillers leads to habituation | M2Q19 | 0.04 | 0.45 |
| M2Q20 | Using painkillers produces good results at my age | M2Q20 | 0.77 | -0.15 |
| M2Q21* | I think that using painkillers causes pain |  | - | - |
| M2Q22 | By using painkillers I will overload my knee/hip | M2Q22 | 0.10 | 0.53 |
| M2Q23** | I have to pay (part of) the costs of painkillers myself |  | 0.05 | 0.18 |
| M2Q24** | More people with OA choose to use painkillers |  | 0.33 | -0.01 |
| M2Q25 | I am scared to use painkillers | M2Q25 | -0.03 | 0.45 |
| M2Q26*** | Painkillers give quick results |  | 0.63 | -0.07 |
| M2Q27** | I think that using painkillers can be repeated |  | 0.45 | -0.17 |
| M2Q28 | I think that using painkillers is invasive | M2Q28 | 0.1 | 0.54 |
| M2Q29*** | I can do my job better by using painkillers |  | 0.76 | 0.06 |
| M2Q30*** | Using painkillers damages my knee/hip |  | -0.02 | 0.45 |
| M2Q31* | I think that using painkillers is easy to perform |  | - | - |
| M2Q32* | My pain lessens by using painkillers |  | - | - |
| M2Q33* | I value the advice of a healthcare provider about using painkillers |  | - | - |
| M2Q34** | I think that using painkillers reduces inflammation |  | 0.35 | 0.03 |
| M2Q35** | By using painkillers, I get something foreign into my body |  | -0.03 | 0.41 |
| M2Q36* | Using painkillers takes a lot of my time |  | - | - |
| M2Q37* | I am dismissive about using painkillers because I believe that OA is a fact of life |  | - | - |

| Module 3: Physiotherapy | | | | |
| --- | --- | --- | --- | --- |
|  | | | Initial factor loadings^ | |
| *Item pool* | *Brief description* | *Retained items* | *Factor I* | *Factor II* |
| M3Q1*** | I think that physiotherapy is painful |  | 0.08 | 0.67 |
| M3Q2** | My thoughts about physiotherapy is: “there’s no harm in trying” |  | 0.07 | 0.11 |
| M3Q3 | My quality of life increases by physiotherapy | M3Q3 | 0.73 | 0.03 |
| M3Q4*** | My pain increases by physiotherapy |  | -0.20 | 0.65 |
| M3Q5*** | I can participate in social activities again by physiotherapy |  | 0.77 | 0.08 |
| M3Q6** | I am more likely to overdo it by physiotherapy |  | 0.29 | 0.55 |
| M3Q7 | I think that physiotherapy involves risks | M3Q7 | -0.10 | 0.58 |
| M3Q8** | The only way to reduce my OA symptoms is by doing physical activities |  | 0.44 | 0.41 |
| M3Q9** | My knee/hip deteriorates faster by physiotherapy |  | -0.26 | 0.46 |
| M3Q10 | I can postpone surgery by physiotherapy | M3Q10 | 0.53 | -0.05 |
| M3Q11*** | I can move more freely by physiotherapy |  | 0.74 | -0.10 |
| M3Q12* | Physiotherapy can be tailored to what I am able to do |  | - | - |
| M3Q13*** | I think it has been proven that physiotherapy reduces OA symptoms |  | 0.61 | -0.04 |
| M3Q14** | I can do physiotherapy together with others |  | 0.35 | 0.04 |
| M3Q15** | I think that physiotherapy involves high costs for society |  | 0.04 | 0.32 |
| M3Q16** | I can do physical activities where and whenever I like |  | 0.21 | 0.01 |
| M3Q17* | Physiotherapy is harmful to my health |  | - | - |
| M3Q18*** | I think that the effect of physiotherapy lasts a long time |  | 0.68 | 0.02 |
| M3Q19** | It is easy to fit physiotherapy into my daily life |  | 0.40 | -0.13 |
| M3Q20 | I can do household chores better by physiotherapy | M3Q20 | 0.86 | 0.06 |
| M3Q21** | I enjoy physiotherapy |  | 0.38 | -0.10 |
| M3Q22 | I need to actively get going with physiotherapy myself | M3Q22 | 0.61 | 0.01 |
| M3Q23 | Doing physiotherapy produces good results at my age | M3Q23 | 0.84 | 0.03 |
| M3Q24 | I think that physiotherapy causes pain | M3Q24 | -0.05 | 0.75 |
| M3Q25 | By physiotherapy I will overload my knee/hip | M3Q25 | -0.06 | 0.70 |
| M3Q26** | I have to pay (part of) the costs of physiotherapy myself |  | -0.06 | 0.02 |
| M3Q27** | More people with OA choose to do physiotherapy |  | 0.35 | 0.04 |
| M3Q28** | I am able to stop taking painkillers by physiotherapy |  | 0.23 | -0.00 |
| M3Q29* | I am scared to do physiotherapy |  | - | - |
| M3Q30** | Physiotherapy takes up my energy |  | 0.11 | 0.35 |
| M3Q31*** | Physiotherapy gives quick results |  | 0.69 | 0.02 |
| M3Q32* | I think that physiotherapy can be repeated |  | - | - |
| M3Q33*** | I learn to deal with my complaints better by physiotherapy |  | 0.72 | -0.06 |
| M3Q34 | I can do my job better by physiotherapy | M3Q34 | 0.80 | -0.05 |
| M3Q35* | Physiotherapy damages my knee/hip |  | - | - |
| M3Q36** | I think that physiotherapy is easy to perform |  | 0.33 | -0.23 |
| M3Q37*** | My pain lessens by physiotherapy |  | 0.76 | -0.17 |
| M3Q38* | I value the advice of a healthcare provider about physiotherapy |  | - | - |
| M3Q39** | I think that physiotherapy reduces inflammation |  | 0.44 | 0.14 |
| M3Q40* | Physiotherapy can be tailored to my goals |  | - | - |
| M3Q41** | Physiotherapy takes a lot of my time |  | 0.00 | 0.20 |
| M3Q42* | I am dismissive about physiotherapy because I believe that OA is a fact of life |  | - | - |

| Module 4: Injections | | | | |
| --- | --- | --- | --- | --- |
| Items | | | Initial factor loadings^ | |
| *Item pool* | *Brief description* | *Retained items* | *Factor I* | *Factor II* |
| M4Q1** | I think that an injection is painful |  | -0.03 | 0.37 |
| M4Q2** | My thoughts about an injection is: “there’s no harm in trying” |  | 0.13 | -0.23 |
| M4Q3 | My quality of life increases by an injection | M4Q3 | 0.78 | -0.09 |
| M4Q4** | My pain increases by an injection |  | -0.30 | 0.35 |
| M4Q5*** | I can participate in social activities again by an injection |  | 0.80 | 0.03 |
| M4Q6** | I am more likely to overdo it by an injection |  | 0.46 | 0.50 |
| M4Q7 | I think that an injection involves risks | M4Q7 | -0.09 | 0.61 |
| M4Q8** | The only option to reduce my OA symptoms is an injection |  | 0.35 | 0.18 |
| M4Q9*** | My knee/hip deteriorates faster by an injection |  | -0.14 | 0.53 |
| M4Q10 | I can postpone surgery by an injection | M4Q10 | 0.65 | 0.18 |
| M4Q11*** | I can move more freely by an injection |  | 0.86 | 0.00 |
| M4Q12*** | I think that an injection has side-effects |  | -0.02 | 0.53 |
| M4Q13*** | I think it has been proven that physical activities reduce OA symptoms |  | 0.57 | -0.10 |
| M4Q14** | I think that an injection involves high costs for society |  | 0.02 | 0.33 |
| M4Q15 | I am becoming dependent on an injection | M4Q15 | 0.19 | 0.60 |
| M4Q16** | I can get an injection where and whenever I like |  | 0.16 | 0.07 |
| M4Q17*** | An injection is harmful to my health |  | -0.07 | 0.54 |
| M4Q18*** | I think that the effect of an injection lasts a long time |  | 0.48 | -0.13 |
| M4Q19** | It is easy to fit an injection into my daily life |  | 0.51 | -0.27 |
| M4Q20 | I can do household chores better by an injection | M4Q20 | 0.86 | -0.03 |
| M4Q21** | I think that an injection leads to habituation |  | 0.24 | 0.53 |
| M4Q22*** | An injection produces good results at my age |  | 0.79 | 0.03 |
| M4Q23** | I think that an injection causes pain |  | -0.21 | 0.46 |
| M4Q24 | By an injection I will overload my knee/hip | M4Q24 | 0.15 | 0.62 |
| M4Q25** | I have to pay (part of) the costs of an injection myself |  | 0.08 | 0.25 |
| M4Q26** | More people with OA choose to do an injection |  | 0.32 | 0.11 |
| M4Q27** | I am able to stop taking painkillers by an injection |  | 0.26 | 0.23 |
| M4Q28*** | I think that an injection carries the chance of an infection |  | -0.12 | 0.55 |
| M4Q29** | I am scared of an injection |  | -0.04 | 0.35 |
| M4Q30 | An injection gives quick results | M4Q30 | 0.80 | -0.01 |
| M4Q31 | I think that an injection can be repeated | M4Q31 | 0.46 | -0.16 |
| M4Q32 | I think that an injection is invasive | M4Q32 | -0.09 | 0.56 |
| M4Q33 | I can do my job better by an injection | M4Q33 | 0.86 | 0.00 |
| M4Q34 | An injection damages my knee/hip | M4Q34 | -0.16 | 0.63 |
| M4Q35** | I think that an injection is easy to perform |  | 0.25 | -0.34 |
| M4Q36*** | My pain lessens by an injection |  | 0.85 | -0.02 |
| M4Q37* | I value the advice of a healthcare provider about an injection |  | - | - |
| M4Q38** | I think an injection reduces inflammation |  | 0.43 | -0.09 |
| M4Q39** | By an injection, I get something foreign into my body |  | -0.10 | 0.42 |
| M4Q40 | An injection takes a lot of my time | M4Q40 | -0.13 | 0.45 |
| M4Q41* | I am dismissive about an injection because I believe that OA is a fact of life |  | - | - |

| Module 5: Arthroplasty | | | | |
| --- | --- | --- | --- | --- |
|  | | | Initial factor loadings^ | |
| *Item pool* | *Brief description* | *Retained items* | *Factor I* | *Factor II* |
| M5Q1 | I think a joint replacement is painful | M5Q1 | -0.10 | 0.63 |
| M5Q2** | My thoughts about joint replacement is: “there’s no harm in trying” |  | -0.05 | -0.13 |
| M5Q3* | My quality of life increases by a joint replacement |  | - | - |
| M5Q4 | My pain increases by a joint replacement | M5Q4 | -0.57 | 0.14 |
| M5Q5*** | I can participate in social activities again by a joint replacement |  | 0.73 | 0.02 |
| M5Q6** | I am more likely to overdo it by a joint replacement |  | -0.03 | 0.23 |
| M5Q7*** | I think that a joint replacement involves risks |  | -0.04 | 0.63 |
| M5Q8** | The only option to decrease my OA symptoms is a joint replacement |  | 0.44 | -0.01 |
| M5Q9 | My knee/hip deteriorates faster by a joint replacement | M5Q9 | -0.46 | 0.11 |
| M5Q10 | I can move more freely after a joint replacement | M5Q10 | 0.83 | 0.04 |
| M5Q11** | I need help and care after a joint replacement |  | 0.15 | 0.40 |
| M5Q12*** | I think it has been proven that a joint replacement reduces OA symptoms |  | 0.55 | 0.15 |
| M5Q13** | I think that a joint replacement involves high costs for society |  | 0.02 | 0.35 |
| M5Q14** | I can do a joint replacement where and whenever I like |  | 0.00 | -0.18 |
| M5Q15** | A joint replacement is harmful to my health |  | -0.32 | 0.25 |
| M5Q16** | It is easy to fit a joint replacement into my daily life |  | 0.29 | -0.20 |
| M5Q17 | I can do household chores better after a joint replacement | M5Q17 | 0.81 | 0.04 |
| M5Q18 | A joint replacement produces good results at my age | M5Q18 | 0.81 | -0.04 |
| M5Q19*** | I think that a joint replacement causes pain |  | -0.09 | 0.60 |
| M5Q20** | I have to pay (part of) the costs of a joint replacement myself |  | -0.15 | 0.13 |
| M5Q21 | More people with OA choose to do a joint replacement | M5Q21 | 0.47 | 0.11 |
| M5Q22** | I am able to stop taking painkillers after a joint replacement |  | 0.42 | -0.03 |
| M5Q23 | I think an artificial joint carries the chance of an infection | M5Q23 | -0.01 | 0.45 |
| M5Q24 | I think a joint replacement carries the chance of an infection | M5Q24 | 0.15 | 0.57 |
| M5Q25** | I am allowed to perform all physicals with an artificial joint |  | 0.25 | -0.04 |
| M5Q26** | I am scared of a joint replacement |  | -0.26 | 0.45 |
| M5Q27 | A joint replacement takes up my energy | M5Q27 | -0.00 | 0.61 |
| M5Q28*** | A joint replacement gives quick results |  | 0.59 | -0.12 |
| M5Q29 | I think that a joint replacement can be repeated | M5Q29 | 0.47 | 0.00 |
| M5Q30 | I think that a joint replacement is invasive | M5Q30 | 0.10 | 0.64 |
| M5Q31*** | I can do my job better by a joint replacement |  | 0.79 | 0.03 |
| M5Q32** | I think a joint replacement is easy to perform |  | 0.39 | -0.28 |
| M5Q33*** | My pain lessens by a joint replacement |  | 0.82 | -0.03 |
| M5Q34* | I value the advice of a healthcare provider about a joint replacement |  | - | - |
| M5Q35** | I think that an artificial joint reduces inflammation |  | 0.35 | 0.07 |
| M5Q36* | With an artificial joint, I get something foreign into my body |  | - | - |
| M5Q37 | A joint replacement takes a lot of my time | M5Q37 | 0.06 | 0.64 |
| M5Q38 | I think that an artificial joint lasts a long time | M5Q38 | 0.48 | 0.22 |
| M5Q39* | I am dismissive about a joint replacement because I believe that OA is a fact of life |  | - | - |
